# Supplementary material for: Scoping Review on Interventions for Physical Activity and Physical Literacy Components in Brazilian School-Aged Children and Adolescents
Source: Int J Environ Res Public Health. 2021 Aug 6;18(16):8349. doi: 10.3390/ijerph18168349 (PMC8392581; doi:10.3390/ijerph18168349)
Supplement: Supplementary file 1 [file ijerph-18-08349-s001.zip › ijerph-1276159-supplementary.pdf]

Supplementary Materials

Table S1. Preferred Reporting Items for Systematic Reviews and Meta-Analyses Extension for Scoping Reviews (Prisma-Scr) Checklist.

SECTION	ITEM	PRISMA-ScR CHECKLIST ITEM	REPORTED ON PAGE #
		**TITLE**	
Title	1	Identify the report as a scoping review.	1
		**ABSTRACT**	
Structured summary	2	Provide a structured summary that includes (as applicable): background, objectives, eligibility criteria, sources of evidence, charting methods, results, and conclusions that relate to the review questions and objectives.	1
		**INTRODUCTION**	
Rationale	3	Describe the rationale for the review in the context of what is already known. Explain why the review questions/objectives lend themselves to a scoping review approach.	2
Objectives	4	Provide an explicit statement of the questions and objectives being addressed with reference to their key elements (e.g., population or participants, concepts, and context) or other relevant key elements used to conceptualize the review questions and/or objectives.	2
		**METHODS**	
Protocol and registration	5	Indicate whether a review protocol exists; state if and where it can be accessed (e.g., a Web address); and, if available, provide registration information, including the registration number.	2
Eligibility criteria	6	Specify characteristics of the sources of evidence used as eligibility criteria (e.g., years considered, language, and publication status), and provide a rationale.	2–3
Information sources	7	Describe all information sources in the search (e.g., databases with dates of coverage and contact with authors to identify additional sources) as well as the date the most recent search was executed.	3
Search	8	Present the full electronic search strategy for at least one database, including any limits used, such that it could be repeated.	Table S3
Selection of sources of evidence †	9	State the process for selecting sources of evidence (i.e., screening and eligibility) included in the scoping review.	3
Data charting process	10	Describe the methods of charting data from the included sources of evidence (e.g., calibrated forms or forms that have been tested by the team before their use, and whether data charting was done independently or in duplicate) and any processes for obtaining and confirming data from investigators.	3
Data items	11	List and define all variables for which data were sought and any assumptions and simplifications made.	3
Critical appraisal of individual sources of evidence	12	If done, provide a rationale for conducting a critical appraisal of included sources of evidence; describe the methods used and how this information was used in any data synthesis (if appropriate).	–
Synthesis of results	13	Describe the methods of handling and summarizing the data that were charted.	4
		**RESULTS**	
Selection of sources of evidence	14	Give numbers of sources of evidence screened, assessed for eligibility, and included in the review, with reasons for exclusions at each stage, ideally using a flow diagram.	4–5
Characteristics of sources of evidence	15	For each source of evidence, present characteristics for which data were charted and provide the citations.	5–9

Critical appraisal within sources of evidence	16	If done, present data on critical appraisal of included sources of evidence (see item 12).	–
Results of individual sources of evidence	17	For each included source of evidence, present the relevant data that were charted that relate to the review questions and objectives.	5–9
Synthesis of results	18	Summarize and/or present the charting results as they relate to the review questions and objectives.	5–9
		**DISCUSSION**	
Summary of evidence	19	Summarize the main results (including an overview of concepts, themes, and types of evidence available), link to the review questions and objectives, and consider the relevance to key groups.	9–11
Limitations	20	Discuss the limitations of the scoping review process.	–
Conclusions	21	Provide a general interpretation of the results with respect to the review questions and objectives, as well as potential implications and/or next steps.	11
		**FUNDING**	
Funding	22	Describe sources of funding for the included sources of evidence, as well as sources of funding for the scoping review. Describe the role of the funders of the scoping review.	12

JBI = Joanna Briggs Institute; PRISMA-ScR = Preferred Reporting Items for Systematic reviews and Meta-Analyses extension for Scoping Reviews.

From: Tricco et al. [14].

Table S2. Searching Results.

Database	Number of Studies Reached on 19 May 2020	Number of Studies Reached on 23 June 2021 (Filters for 2020 and 2021 Studies)	Searches Combined
Web of Science	577	168	745
EMBASE	136	47	183
ERIC	145	0	145
Medline/PUBMED	324	23	347
PSYCHINFO	254	24	278
SPORTDISCUS	159	17	176
SCOPUS	220	54	274
LILACS	706	25	731
SCIELO	335	47	382
All databases	2856	405	3261
BDTD	148	4	152
Scholar Google	200	100	300
**All (database + gray literature)**	**3204**	**509**	**3713**
**Title for screening process (excluding duplicates)**	**2643**	**301**	**2944**

Table S3. Searching Strategy Implemented at Medline (Pubmed Portal).

Groups of Descriptors	Descriptors	Number of Studies Reached on 19 May 2020	Number of Studies Reached on 23 June 2021 (Filters for 2020 and 2021 Studies)
**#1–PA-Attributes**	**"Physical Fitness"[MeSH Terms] OR "Exercise Test"[MeSH Terms] OR "Physical Fitness"[Text Word] OR "Fitness Testing"[Text Word] OR "Fitness Tests"[Text Word] OR "Cardiorespiratory Fitness" [MeSH Terms] OR "Aerobic Fitness"[Text Word] OR "Aerobic Capacity"[Text Word] OR "Maximum Oxygen Consumption"[Text Word] OR"Maximal Oxygen Uptake"[Text Word] OR "Cardiovascular Fitness"[Text Word] OR Pulse Rate[Text Word] OR Heart Rate[MeSH Terms] OR "Muscle Strength"[MeSH Terms] OR "Muscular Fitness"[Text Word] OR "Muscle Contraction"[MeSH Terms] OR "Muscular Strength"[Text Word] OR "Muscular Endurance"[Text Word] OR "Muscular Power"[Text Word] OR "Lower Body Explosive Strength"[Text Word] OR "Lower Limb Strength"[Text Word] OR "Upper Limb Strength"[Text Word] OR "Upper Body Strength"[Text Word] OR "Lower Body Strength"[Text Word] OR "Musculoskeletal Fitness"[Text Word] OR "Isometric Strength"[Text Word] OR "Dynamic Strength"[Text Word] OR "Isometric Contraction"[MeSH Terms] OR "Isotonic Contraction"[MeSH Terms] OR "Dynamic Force"[Text Word] OR Flexibility[Text Word] OR Pliability[MeSH Terms] OR "Body Composition"[MeSH Terms] OR "Body Composition"[Text Word] OR "Body Fat Distribution"[Mesh Terms] OR "Adiposity"[Mesh Terms] OR "Adiposity"[Text Word] OR "Body Mass Index"[Mesh Terms] OR "Body Mass Index"[Text Word] OR BMI[Text Word] OR Obesity[Text Word] OR Obesity[Mesh Terms] OR obese[Text Word] OR overweight[Text Word] OR "weight excess"[Text Word] OR "body fat"[Text Word] OR "fat mass" [Text Word] OR "fat free mass" [Text Word] OR "lean mass" [Text Word] OR Skinfold[Text Word] OR Waist circumference[Text Word] OR Antropometr*[Text Word] OR ((movement[Text Word]) AND (skills[Text Word] OR control[Text Word] OR repertoire[Text Word] OR pattern*[Text Word] OR vocabulary[Text Word] OR competence*[Text Word] OR capacity*[Text Word] OR capabilit*[Text Word] OR performance[Text Word])) OR ((Motor[Text Word]) AND (abilit*[Text Word] OR development[Text Word] OR performance[Text Word] OR control[Text Word] OR coordination[Text Word] OR skills[Text Word] OR Proficiency[Text Word] OR competence*[Text Word] OR acquisition[Text Word])) OR "Physical Literacy"[Text Word] OR "Motor Skills"[MeSH Terms]**	**1,368,721**	**128,063**
**#2–PA-Associated Factors**	**(Attitudes[MeSH Terms] OR Attitude*[Text Word] OR Motivation[MeSH Terms] OR Motivation[Text Word] OR "Self Concept"[MeSH Terms] OR knowledge[MeSH Terms] OR knowledge[Text Word] OR "Self Efficacy"[MeSH Terms] OR "Self Efficacy"[Text Word] OR "Self Perception"[Text Word] OR "Competence"[Text Word] OR "Self Esteem"[Text Word] OR "Self Regulation"[Text Word] OR emotion*[Text Word] OR awareness[Text Word] OR Satisfaction[Text Word] OR Enjoyment[Text Word] OR Enjoy[Text Word] OR Feeling*[Text Word] OR "Self Control"[Text Word] OR Benefit*[Text Word] OR Barrier*[Text Word] OR opportunit*[Text Word] OR experienc*[Text Word] OR Engagement[Text Word] OR Enjoyment[Text Word] OR value*[Text Word] OR understanding[Text Word] OR responsibility[Text Word] OR proficiency[Text Word] OR ((Motivational[Text Word] OR Affective[Text Word] OR emotional[Text Word]**	**452,634**	**49,403**

	OR Psychologic*[Text Word] OR Psychosocial[Text Word] OR Cognitive[Text Word] OR physical[Text Word] OR social[Text Word]) AND (capacity[Text Word] OR capacities[Text Word] OR capabilit*[Text Word] OR determinant*[Text Word] OR correlate*[Text Word] OR Mediator*[Text Word] OR factor*[Text Word])) OR ((Cultural[Text Word] OR Social[Text Word] OR Sociocultural[Text Word] OR peer*[Text Word] OR family[Text Word] OR parents[Text Word] OR friend*[Text Word]) AND (Support[Text Word] OR Modelling[Text Word] OR Norm*[Text Word] OR Rule*[Text Word])) OR "Social Support"[MeSH Terms] OR "Social Norms"[MeSH Terms] OR Connectedness[Text Word] OR "Community Participation"[MeSH Terms] OR "Social Environment"[MeSH Terms] OR "Outdoor Environment"[Text Word] OR "Environment Design" [MeSH Terms] OR "Physical Environment"[Text Word] OR "Environmental Health"[MeSH Terms] OR "School Environment"[Text Word] OR "Recreational Environment"[Text Word] OR Walkability[Text Word] OR neighborhood*[Text Word] OR community[Text Word] OR Safety[MeSH Terms]) AND ("Motor Activity"[MeSH Terms] OR "Physical Activity"[Text Word] OR Physical Activiti*[Text Word] OR Exercise[MeSH Terms] OR "Exercise"[Text Word] OR Exercise*[Text Word] OR Sports[MeSH Terms] OR Sport*[Text Word] OR Movement[Text Word] OR Physical Education[Text Word])		
#3–PA-Behavior	"Motor Activity"[MeSH Terms] OR "Physical Activity"[Text Word] OR Physical Activiti*[Text Word] OR Exercise[MeSH Terms] OR "Exercise"[Text Word] OR Exercise*[Text Word] OR Sports[MeSH Terms] OR Sport*[Text Word] OR Danc*[Text Word] OR Walking[MeSH Terms] OR Bicycling[MeSH Terms] OR "Active Commuting"[Text Word] OR "Active Transport"[Text Word] OR Physical Education and Training[MeSH Terms] OR Physical Education[Text Word] OR "Leisure Activities"[MeSH Terms] OR Recreation[Text Word] OR "play and playthings"[MeSH Terms] OR "play time"[Text Word] OR "playing" [Text Word] OR Acceleromet*[Text Word] OR pedomet*[Text Word] OR inclinomet*[Text Word]	852,060	76,938
#4<br><br>#1 OR #2 OR #3	(("Physical Fitness"[MeSH Terms] OR "Exercise Test"[MeSH Terms] OR "Physical Fitness"[Text Word] OR "Fitness Testing"[Text Word] OR "Fitness Tests"[Text Word] OR "Cardiorespiratory Fitness" [MeSH Terms] OR "Aerobic Fitness"[Text Word] OR "Aerobic Capacity"[Text Word] OR "Maximum Oxygen Consumption"[Text Word] OR"Maximal Oxygen Uptake"[Text Word] OR "Cardiovascular Fitness"[Text Word] OR Pulse Rate[Text Word] OR Heart Rate[MeSH Terms] OR "Muscle Strength"[MeSH Terms] OR "Muscular Fitness"[Text Word] OR "Muscle Contraction"[MeSH Terms] OR "Muscular Strength"[Text Word] OR "Muscular Endurance"[Text Word] OR "Muscular Power"[Text Word] OR "Lower Body Explosive Strength"[Text Word] OR "Lower Limb Strength"[Text Word] OR "Upper Limb Strength"[Text Word] OR "Upper Body Strength"[Text Word] OR "Lower Body Strength"[Text Word] OR "Musculoskeletal Fitness"[Text Word] OR "Isometric Strength"[Text Word] OR "Dynamic Strength"[Text Word] OR "Isometric Contraction"[MeSH Terms] OR "Isotonic Contraction"[MeSH Terms] OR "Dynamic Force"[Text Word] OR Flexibility[Text Word] OR Pliability[MeSH Terms] OR "Body Composition"[MeSH Terms] OR "Body Composition"[Text Word] OR "Body Fat Distribution"[Mesh Terms] OR "Adiposity"[Mesh Terms] OR "Adiposity"[Text Word] OR "Body Mass Index"[Mesh Terms] OR "Body Mass Index"[Text Word] OR BMI[Text Word] OR Obesity[Text Word] OR Obesity[Mesh Terms] OR obese[Text Word] OR overweight[Text Word] OR "weight excess"[Text Word] OR "body fat"[Text Word] OR "fat mass" [Text Word]	1,980,515	187,730

OR “fat free mass” [Text Word] OR “lean mass” [Text Word] OR Skinfold[Text Word] OR Waist circumference[Text Word] OR Antropometr*[Text Word] OR ((movement[Text Word]) AND (skills[Text Word] OR control[Text Word] OR repertoire[Text Word] OR pattern*[Text Word] OR vocabulary[Text Word] OR competence*[Text Word] OR capacity*[Text Word] OR capabilit*[Text Word] OR performance[Text Word])) OR ((Motor[Text Word]) AND (abilit*[Text Word] OR development[Text Word] OR performance[Text Word] OR control[Text Word] OR coordination[Text Word] OR skills[Text Word] OR Proficiency[Text Word] OR competence*[Text Word] OR acquisition[Text Word])) OR “Physical Literacy”[Text Word] OR “Motor Skills”[MeSH Terms]) OR ((Attitudes[MeSH Terms] OR Attitude*[Text Word] OR Motivation[MeSH Terms] OR Motivation[Text Word] OR “Self Concept”[MeSH Terms] OR knowledge[MeSH Terms] OR knowledge[Text Word] OR “Self Efficacy”[MeSH Terms] OR “Self Efficacy”[Text Word] OR “Self Perception”[Text Word] OR “Competence”[Text Word] OR “Self Esteem”[Text Word] OR “Self Regulation”[Text Word] OR emotion*[Text Word] OR awareness[Text Word] OR Satisfaction[Text Word] OR Enjoyment[Text Word] OR Enjoy[Text Word] OR Feeling*[Text Word] OR “Self Control”[Text Word] OR Benefit*[Text Word] OR Barrier*[Text Word] OR opportunit*[Text Word] OR experienc*[Text Word] OR Engagement[Text Word] OR Enjoyment[Text Word] OR value*[Text Word] OR understanding[Text Word] OR responsibility[Text Word] OR proficiency[Text Word] OR ((Motivational[Text Word] OR Affective[Text Word] OR emotional[Text Word] OR Psychologic*[Text Word] OR Psychosocial[Text Word] OR Cognitive[Text Word] OR physical[Text Word] OR social[Text Word]) AND (capacity[Text Word] OR capacities[Text Word] OR capabilit*[Text Word] OR determinant*[Text Word] OR correlate*[Text Word] OR Mediator*[Text Word] OR factor*[Text Word])) OR ((Cultural[Text Word] OR Social[Text Word] OR Sociocultural[Text Word] OR peer*[Text Word] OR family[Text Word] OR parents[Text Word] OR friend*[Text Word]) AND (Support[Text Word] OR Modelling[Text Word] OR Norm*[Text Word] OR Rule*[Text Word])) OR “Social Support”[MeSH Terms] OR “Social Norms”[MeSH Terms] OR Connectedness[Text Word] OR “Community Participation”[MeSH Terms] OR “Social Environment”[MeSH Terms] OR “Outdoor Environment”[Text Word] OR “Environment Design” [MeSH Terms] OR “Physical Environment”[Text Word] OR “Environmental Health”[MeSH Terms] OR “School Environment”[Text Word] OR “Recreational Environment”[Text Word] OR Walkability[Text Word] OR neighborhood*[Text Word] OR community[Text Word] OR Safety[MeSH Terms]) AND (“Motor Activity”[MeSH Terms] OR “Physical Activity”[Text Word] OR Physical Activiti*[Text Word] OR Exercise[MeSH Terms] OR "Exercise"[Text Word] OR Exercise*[Text Word] OR Sports[MeSH Terms] OR Sport*[Text Word] OR Movement[Text Word] OR Physical Education[Text Word]))) OR (“Motor Activity”[MeSH Terms] OR “Physical Activity”[Text Word] OR Physical Activiti*[Text Word] OR Exercise[MeSH Terms] OR "Exercise"[Text Word] OR Exercise*[Text Word] OR Sports[MeSH Terms] OR Sport*[Text Word] OR Danc*[Text Word] OR Walking[MeSH Terms] OR Bicycling[MeSH Terms] OR “Active Commuting”[Text Word] OR “Active Transport”[Text Word] OR Physical Education and Training[MeSH Terms] OR Physical Education[Text Word] OR “Leisure Activities”[MeSH Terms] OR Recreation[Text Word] OR “play and playthings"[MeSH Terms] OR "play time"[Text Word] OR "playing" [Text Word] OR Acceleromet*[Text Word] OR pedomet*[Text Word] OR inclinomet*[Text Word])

**#5–Context**	**Brazil[MeSH Terms] OR Brazil[Text Word] OR Brazilian*[Text Word]**	**129,686**	**17,985**
**#6–Population**	**child[MeSH Terms] OR adolescent[MeSH Terms] OR students[MeSH Terms] OR student[Text Word] OR students[Text Word] OR "young children"[Text Word] OR "young people"[Text Word] OR children[Text Word] OR adolescent[Text Word] OR adolescents[Text Word] OR young[Text Word] OR pupils[Text Word] OR Schoolage*[Text Word] OR schoolchild*[Text Word] OR schooler*[Text Word]**	**4,124,092**	**278,809**
**#7–Study Design**	**(“controlled clinical trial"[Publication Type] OR "controlled clinical trials as topic"[MeSH Terms] OR "controlled clinical trial"[All Fields] OR “randomized controlled trial”[Publication Type] OR "randomized controlled trials as topic"[MeSH Terms] OR “random allocation”[MeSH Terms] OR “double-blind method”[MeSH Terms] OR “single-blind method”[MeSH Terms] OR “double blind study”[text word] OR “single blind study”[text word] OR “triple blind study”[text word] OR (clinical* [Text Word] AND trial* [Text Word] OR ((randomised[Text Word] OR randomized[Text Word] OR randomly[Text Word]) AND (trial*[Text Word] OR group*[Text Word])) OR trial*[Title] OR “non-randomized controlled trial”[text word] OR "non-randomized controlled trials as topic"[MeSH Terms] OR “nonrandomized controlled trial”[text word] OR “non-randomized trial”[text word] OR “intervention study”[text word] OR “intervention studies”[text word] OR “intervention program”[text word] OR "intervention trial"[text word] OR "Comparative Study"[Publication Type] OR "Evaluation Study"[Publication Type] OR “follow-up studies”[MeSH Terms] OR “prospective studies”[MeSH Terms] OR "longitudinal studies"[MeSH Terms] OR “cross-over studies”[MeSH Terms] OR “interrupted time series analysis”[MeSH Terms] OR “quasiexperimental”[Text Word]) OR “quasi experimental”[Text Word] OR “pseudo experimental”[Text Word] OR "Before-After Studies"[text word] OR "Before-After Study"[text word] OR “Controlled Before-After Studies”[MeSH Terms] OR “Outcome Assessment, Health Care"[MeSH Terms])**	**4,866,388**	**288,343**
**#8**<br><br>**#4 AND #5 AND #6 AND #7**<br><br>**Using filters for intervention studies**	Search: **((((“controlled clinical trial"[Publication Type] OR "controlled clinical trials as topic"[MeSH Terms] OR "controlled clinical trial"[All Fields] OR “randomized controlled trial”[Publication Type] OR "randomized controlled trials as topic"[MeSH Terms] OR “random allocation”[MeSH Terms] OR “double-blind method”[MeSH Terms] OR “single-blind method”[MeSH Terms] OR “double blind study”[text word] OR “single blind study”[text word] OR “triple blind study”[text word] OR (clinical* [Text Word] AND trial* [Text Word] OR ((randomised[Text Word] OR randomized[Text Word] OR randomly[Text Word]) AND (trial*[Text Word] OR group*[Text Word])) OR trial*[Title] OR “non-randomized controlled trial”[text word] OR "non-randomized controlled trials as topic"[MeSH Terms] OR “nonrandomized controlled trial”[text word] OR “non-randomized trial”[text word] OR “intervention study”[text word] OR “intervention studies”[text word] OR “intervention program”[text word] OR "intervention trial"[text word] OR "Comparative Study"[Publication Type] OR "Evaluation Study"[Publication Type] OR “follow-up studies”[MeSH Terms] OR “prospective studies”[MeSH Terms] OR "longitudinal studies"[MeSH Terms] OR “cross-over studies”[MeSH Terms] OR “interrupted time series analysis”[MeSH Terms] OR “quasiexperimental”[Text Word]) OR “quasi experimental”[Text Word] OR “pseudo experimental”[Text Word] OR "Before-After Studies"[text word] OR "Before-After Study"[text word] OR “Controlled Before-After Studies”[MeSH Terms] OR “Outcome Assessment, Health Care"[MeSH Terms])) AND (child[MeSH Terms] OR adolescent[MeSH Terms] OR students[MeSH Terms] OR student[Text Word] OR students[Text Word]**	**324**	**23**

OR "young children"[Text Word] OR "young people"[Text Word] OR children[Text Word] OR adolescent[Text Word] OR adolescents[Text Word] OR young[Text Word] OR pupils[Text Word] OR Schoolage*[Text Word] OR schoolchild*[Text Word] OR schooler*[Text Word])) AND (Brazil[MeSH Terms] OR Brazil[Text Word] OR Brazilian*[Text Word])) AND (((“Physical Fitness”[MeSH Terms] OR “Exercise Test”[MeSH Terms] OR “Physical Fitness”[Text Word] OR “Fitness Testing”[Text Word] OR “Fitness Tests”[Text Word] OR “Cardiorespiratory Fitness” [MeSH Terms] OR “Aerobic Fitness”[Text Word] OR “Aerobic Capacity”[Text Word] OR “Maximum Oxygen Consumption”[Text Word] OR“Maximal Oxygen Uptake”[Text Word] OR “Cardiovascular Fitness”[Text Word] OR Pulse Rate[Text Word] OR Heart Rate[MeSH Terms] OR “Muscle Strength”[MeSH Terms] OR “Muscular Fitness”[Text Word] OR “Muscle Contraction”[MeSH Terms] OR “Muscular Strength”[Text Word] OR “Muscular Endurance”[Text Word] OR “Muscular Power”[Text Word] OR “Lower Body Explosive Strength”[Text Word] OR “Lower Limb Strength”[Text Word] OR “Upper Limb Strength”[Text Word] OR “Upper Body Strength”[Text Word] OR “Lower Body Strength”[Text Word] OR “Musculoskeletal Fitness”[Text Word] OR “Isometric Strength”[Text Word] OR “Dynamic Strength”[Text Word] OR “Isometric Contraction”[MeSH Terms] OR “Isotonic Contraction”[MeSH Terms] OR “Dynamic Force”[Text Word] OR Flexibility[Text Word] OR Pliability[MeSH Terms] OR “Body Composition”[MeSH Terms] OR “Body Composition”[Text Word] OR "Body Fat Distribution"[Mesh Terms] OR "Adiposity"[Mesh Terms] OR "Adiposity"[Text Word] OR "Body Mass Index"[Mesh Terms] OR "Body Mass Index"[Text Word] OR BMI[Text Word] OR Obesity[Text Word] OR Obesity[Mesh Terms] OR obese[Text Word] OR overweight[Text Word] OR “weight excess”[Text Word] OR “body fat”[Text Word] OR “fat mass” [Text Word] OR “fat free mass” [Text Word] OR “lean mass” [Text Word] OR Skinfold[Text Word] OR Waist circumference[Text Word] OR Antropometr*[Text Word] OR ((movement[Text Word]) AND (skills[Text Word] OR control[Text Word] OR repertoire[Text Word] OR pattern*[Text Word] OR vocabulary[Text Word] OR competence*[Text Word] OR capacity*[Text Word] OR capabilit*[Text Word] OR performance[Text Word])) OR ((Motor[Text Word]) AND (abilit*[Text Word] OR development[Text Word] OR performance[Text Word] OR control[Text Word] OR coordination[Text Word] OR skills[Text Word] OR Proficiency[Text Word] OR competence*[Text Word] OR acquisition[Text Word])) OR “Physical Literacy”[Text Word] OR “Motor Skills”[MeSH Terms]) OR ((Attitudes[MeSH Terms] OR Attitude*[Text Word] OR Motivation[MeSH Terms] OR Motivation[Text Word] OR “Self Concept”[MeSH Terms] OR knowledge[MeSH Terms] OR knowledge[Text Word] OR “Self Efficacy”[MeSH Terms] OR “Self Efficacy”[Text Word] OR “Self Perception”[Text Word] OR “Competence”[Text Word] OR “Self Esteem”[Text Word] OR “Self Regulation”[Text Word] OR emotion*[Text Word] OR awareness[Text Word] OR Satisfaction[Text Word] OR Enjoyment[Text Word] OR Enjoy[Text Word] OR Feeling*[Text Word] OR “Self Control”[Text Word] OR Benefit*[Text Word] OR Barrier*[Text Word] OR opportunit*[Text Word] OR experienc*[Text Word] OR Engagement[Text Word] OR Enjoyment[Text Word] OR value*[Text Word] OR understanding[Text Word] OR responsibility[Text Word] OR proficiency[Text Word] OR ((Motivational[Text Word] OR Affective[Text Word] OR emotional[Text Word] OR Psychologic*[Text Word] OR Psychosocial[Text Word] OR Cognitive[Text Word] OR physical[Text Word] OR social[Text Word]) AND (capacity[Text Word] OR ca-

pacities[Text Word] OR capabilit*[Text Word] OR determinant*[Text Word] OR correlate*[Text Word] OR Mediator*[Text Word] OR factor*[Text Word])) OR ((Cultural[Text Word] OR Social[Text Word] OR Soci-ocultural[Text Word] OR peer*[Text Word] OR family[Text Word] OR parents[Text Word] OR friend*[Text Word]) AND (Support[Text Word] OR Modelling[Text Word] OR Norm*[Text Word] OR Rule*[Text Word])) OR "Social Support"[MeSH Terms] OR "Social Norms"[MeSH Terms] OR Connectedness[Text Word] OR "Community Participation"[MeSH Terms] OR "Social Environment"[MeSH Terms] OR "Out-door Environment"[Text Word] OR "Environment Design" [MeSH Terms] OR "Physical Environ-ment"[Text Word] OR "Environmental Health"[MeSH Terms] OR "School Environment"[Text Word] OR "Recreational Environment"[Text Word] OR Walkability[Text Word] OR neighborhood*[Text Word] OR community[Text Word] OR Safety[MeSH Terms]) AND ("Motor Activity"[MeSH Terms] OR "Physical Activity"[Text Word] OR Physical Activiti*[Text Word] OR Exercise[MeSH Terms] OR "Exercise"[Text Word] OR Exercise*[Text Word] OR Sports[MeSH Terms] OR Sport*[Text Word] OR Movement[Text Word] OR Physical Education[Text Word]))) OR ("Motor Activity"[MeSH Terms] OR "Physical Activi-ty"[Text Word] OR Physical Activiti*[Text Word] OR Exercise[MeSH Terms] OR "Exercise"[Text Word] OR Exercise*[Text Word] OR Sports[MeSH Terms] OR Sport*[Text Word] OR Danc*[Text Word] OR Walk-ing[MeSH Terms] OR Bicycling[MeSH Terms] OR "Active Commuting"[Text Word] OR "Active Transport"[Text Word] OR Physical Education and Training[MeSH Terms] OR Physical Education[Text Word] OR "Leisure Activities"[MeSH Terms] OR Recreation[Text Word] OR "play and playthings"[MeSH Terms] OR "play time"[Text Word] OR "playing" [Text Word] OR Acceleromet*[Text Word] OR pedom-et*[Text Word] OR inclinomet*[Text Word])) Filters: **Clinical Study, Clinical Trial**

Table S4. Searching Strategy Implemented at Gray Literature Databases.

Groups of Descriptors	Descriptors
	**Brazilian Digital Library of Theses and Dissertations (BDTD).**
**#1–PA-Attributes**	("Physical Fitness" OR "Cardiorespiratory Fitness" OR "Aerobic Fitness" OR "Cardiovascular Fitness" OR "Muscular Fitness" OR "Muscular Strength" OR "Muscular Endurance" OR Flexibility OR "Body Composition" OR Adiposity OR "Body Mass Index" OR Obesity OR Overweight OR "Weight Excess" OR Antropometr* OR "Physical Literacy" OR "Motor Skills" OR "Movement Skills" OR "Movement Pattern" OR "Movement Performance" OR "Motor Performance" OR "Motor Competence")
**#2–PA-Behavior**	("Physical Activity" OR Exercise OR Sports OR "Physical Education" OR "Leisure Activities" OR Recreation OR Playing OR Acceleromet* OR Pedomet* OR Inclinomet*)
**#3–<br>#1 AND #2**	("Physical Fitness" OR "Cardiorespiratory Fitness" OR "Aerobic Fitness" OR "Cardiovascular Fitness" OR "Muscular Fitness" OR "Muscular Strength" OR "Muscular Endurance" OR Flexibility OR "Body Composition" OR Adiposity OR "Body Mass Index" OR Obesity OR Overweight OR "Weight Excess" OR Antropometr* OR "Physical Literacy" OR "Motor Skills" OR "Movement Skills" OR "Movement Pattern" OR "Movement Performance" OR "Motor Performance" OR "Motor Competence") OR ("Physical Activity" OR Exercise OR Sports OR "Physical Education" OR "Leisure Activities" OR Recreation OR Playing OR Acceleromet* OR Pedomet* OR Inclinomet*)
**#4–Context**	(Brazil*)
**#5–Population**	(Child* OR Adolescent* OR Adolescence* OR Student* OR Young OR Schoolchild*)
**#6–Study Design**	Title:(Trial* OR Intervention* OR Program* OR Experiment* OR "Clinical Study")
**#7–#3 AND #4 AND #5 AND #6**	(All Fields:("Physical Fitness" OR "Cardiorespiratory Fitness" OR "Aerobic Fitness" OR "Cardiovascular Fitness" OR "Muscular Fitness" OR "Muscular Strength" OR "Muscular Endurance" OR Flexibility OR "Body Composition" OR Adiposity OR "Body Mass Index" OR Obesity OR Overweight OR "Weight Excess" OR Antropometr* OR "Physical Literacy" OR "Motor Skills" OR "Movement Skills" OR "Movement Pattern" OR "Movement Performance" OR "Motor Performance" OR "Motor Competence") OR ("Physical Activity" OR Exercise OR Sports OR "Physical Education" OR "Leisure Activities" OR Recreation OR Playing OR Acceleromet* OR Pedomet* OR Inclinomet*)) AND (All Fields:(Brazil*)) AND (All Fields:(Child* OR Adolescent* OR Adolescence* OR Student* OR Young OR Schoolchild*)) AND (Title:(Trial* OR Intervention* OR Program* OR Experiment* OR "Clinical Study"))
	**Google Scholar**
**#1**	("Physical Fitness" OR "Cardiorespiratory Fitness" OR "Body Composition" OR "Body Mass Index" OR "Physical Literacy" OR "Motor Skills" OR "Physical Activity" OR "Physical Education")+(Brazil*)+(Student*)+(Trial* OR Intervention* OR Program* OR Experiment*)
	**Brazilian Society on Physical Activity and Health (SBAFS)**
**#1**	(intervenção OR programa) + (“atividade física” OR exercício OR esporte OR “aptidão física” OR “habilidade motora”) + escola
	**Sport Brazil Project (*Projeto Esporte Brasil, PROESP-BR*)**
**#1**	(intervenção OR programa) + (“atividade física” OR exercício OR esporte OR “aptidão física” OR “habilidade motora”) + escola
